# Oxidation-Resistant Coatings for TiAl Intermetallics—Short Review

**DOI:** 10.3390/ma19071408

**Published:** 2026-04-01

**Authors:** Marek Góral, Krzysztof Szymkiewicz

**Affiliations:** 1 Research and Development Laboratory for Aerospace Materials, Rzeszow University of Technology, 35-959 Rzeszów, Poland; mgoral@prz.edu.pl; 2 Faculty of Mechanical Engineering, Cracow University of Technology, 31-155 Kraków, Poland

**Keywords:** TiAl intermetallics, aluminide coating, silicide coatings, oxidation, protective coatings

## Abstract

This article presents a review of current trends in the development of protective coatings for TiAl alloys. These materials have been indicated as potential replacements for nickel-based alloys for several decades; however, many problems related to their application have not yet been solved, such as low resistance to oxidation and high-temperature corrosion, limited wear and erosion resistance, as well as susceptibility to microstructural changes under demanding conditions. Research in this area has been conducted for many years; nevertheless, new concepts and types of coatings are constantly being developed, particularly for third- and fourth-generation TiAl alloys. This article presents a review and classification of protective coatings used for TiAl alloys, with particular emphasis on research results published in the scientific literature of the last decade. In addition to a literature review concerning coating types and applied technologies, new trends and concepts in this field proposed by the co-authors will also be presented.

## 1. Introduction

Intermetallic alloys based on the γ-TiAl phase have been the subject of intensive research for several decades due to their unique combination of low density, high specific strength, and good structural stability at elevated temperatures. They are produced based on intermetallic phases with an ordered structure over a wide temperature range, up to the melting point. Their properties make them dedicated to operation at high temperatures. Hence, they belong to the class of promising structural materials for the aerospace, automotive, and power generation industries [1,2,3]. However, the application of these alloys in components operating in the temperature range of 700–900 °C, such as low-pressure turbine blades or exhaust system elements, is significantly limited by their insufficient resistance to oxidation and high-temperature corrosion [4,5,6].

The second group of materials intended for applications with higher requirements are nickel-based superalloys. However, due to their relatively high density and lower strength, TiAl or NiAl intermetallics serve as substitute materials for conventional nickel superalloys for the production of aerospace components, including gas turbine blades, exhaust system elements, and aircraft turbine parts. Their application allows for a reduction in the mass of the hot part of a gas engine by up to 20–30%, and this can improve the performance and energy efficiency of aircrafts.

The most well-known intermetallics are materials from the NiAl group, intended for operation at higher temperatures. They exhibit good mechanical properties, high melting points, and resistance to elevated temperatures. Moreover, they are characterized by high corrosion resistance, resulting from the presence of a thin protective layer composed of aluminum oxide (Al_2_O_3_) forming on the alloy surface. Nevertheless, although NiAl intermetallics can operate at higher temperatures than TiAl materials, the density of the latter is lower. TiAl intermetallics seem to be a better solution for the mentioned applications; however, they still exhibit certain limitations.

The fundamental problem of γ-TiAl intermetallic alloys in oxidizing environments is their tendency to form a non-protective, porous scale dominated by rutile TiO_2_, which is characterized by high permeability to oxygen and low adhesion to the substrate [7,8]. Although the presence of aluminum potentially enables the formation of a protective Al_2_O_3_ layer, its formation is hindered by rapid diffusion of titanium towards the surface and unfavorable nucleation kinetics of α-Al_2_O_3_ in the initial stages of oxidation [9,10,11]. Consequently, long-term exposure of TiAl alloys to high temperatures leads to accelerated surface degradation, scale cracking, and intensive material loss [12,13,14].

One of the fundamental directions for improving the oxidation resistance of γ-TiAl alloys is the modification of their chemical composition through the introduction of alloying additions, particularly refractory elements such as Nb, Ta, W, and Mo [15,16,17,18]. These elements influence both the thermodynamics and kinetics of oxidation processes, promoting the formation of a continuous and compact Al_2_O_3_ layer and limiting oxygen diffusion into the material [19,20,21,22]. Additionally, reactive elements (Y and Gd) as well as silicon and chromium additions also exhibit a beneficial effect, which can significantly improve the adhesion and mechanical integrity of the scale through the formation of barrier phases or modification of the scale structure [23,24,25,26,27].

A good and promising solution used to protect TiAl alloys against high-temperature degradation is the application of surface treatment processes, leading to the formation of protective surface layers. The aim is to enforce a favorable oxidation mechanism by enriching the surface with aluminum or silicon [28,29,30,31].

Among the most commonly used solutions are simple aluminide coatings produced by diffusion methods (pack cementation, CVD, and slurry), TiAlCr-type coatings, and advanced multicomponent TiAlCrY and TiAlSiN systems, which enable the stable formation of an α-Al_2_O_3_ layer, even at temperatures exceeding 1000 °C [32,33,34,35,36]. Additionally, a significant improvement in oxidation resistance is demonstrated by the application of the so-called halogen effect, consisting of the selective transport of aluminum in the form of volatile halides and its re-oxidation within the scale [37,38,39].

In recent years, a dynamic development of silicon and silicide-based coatings has also been observed, whose protective mechanism is based on the formation of stable SiO_2_ layers or Al_2_O_3_-SiO_2_ mixtures, characterized by very low growth rates and high diffusion tightness [40,41,42,43]. These solutions, in combination with thermal barrier coating (TBC) systems, enable further shifting of the operating temperature limit of γ-TiAl alloys; however, their durability is still determined by the stability of the interdiffusion zone and resistance to cyclic thermal loads [44,45,46].

Research indicates that an appropriately optimized layer deposition process guarantees the attainment of high-quality coatings, effectively mitigating core material degradation and improving erosion resistance and surface wear resistance of the TiAl alloy. In recent years, there has been great interest in surface process technologies and research into new types of coatings, as well as those built from multiple layers or hybrid structures. Many of the proposed technological and material solutions are directed towards third- and fourth-generation TiAl alloys, which exhibit high mechanical properties and resistance to creep processes at elevated temperatures. Many issues related to the improvement of surface properties of intermetallic alloys at elevated temperatures are still not fully understood and explained. Further development of scientific achievements in this area is not possible without a comprehensive literature review, categorization of coatings, and thorough verification of their production methods.

Therefore, the aim of this work is to provide a critical review of the mechanisms for improving the oxidation resistance of γ-TiAl alloys through alloying additions and the application of protective coatings, with particular emphasis on the role of refractory elements, silicon, and the halogen effect. Furthermore, the last part of this work describes current research directions of advanced coating systems that allow for effective protection of TiAl alloys under conditions of elevated temperatures, including environments containing corrosive salts and water vapor. The presented work has been divided into three main parts, covering the classification of coating deposition methods used for TiAl intermetallic alloys, the characteristics of protective coatings, and directions for further research.

## 2. Deposition Methods of Protective Coatings on TiAl Intermetallics

The production of protective coatings on the surface of intermetallic alloys improves their resistance to oxidation and corrosion. The structure and properties of coatings produced on intermetallic alloys depend on the properties of the alloy surface, the type of coating material, and the deposition technique. Therefore, proper preparation of the substrate surface and appropriate selection of coating deposition process parameters are crucial.

Various coating production techniques are used for the surface protection of TiAl alloys, including magnetron sputtering, PVD, CVD, plasma spraying, laser cladding, enameling, the sol–gel method, liquid phase crystallization, ion implantation, and dip coating. Most of these methods enable the production of layers with high adhesion to the substrate and allow the process to be carried out at relatively low temperatures. The most commonly used method is plasma spraying, which allows for the deposition of relatively thick coatings and the use of a wide range of spray materials. A comparison of the advantages and disadvantages of the applied coating deposition processes is presented in Table 1.

## 3. Improvement of Heat Resistance of TiAl Alloys Through Alloying Additives

Refractory elements, such as niobium, tantalum, and tungsten, are recognized as one of the most effective alloying additions that improve the oxidation resistance of TiAl alloys. Their interaction relies on the ability to modify the growth kinetics of the oxide scale and stabilize phases that thermodynamically and kinetically promote the formation of a continuous Al_2_O_3_ layer [2,3].

The beneficial effects of niobium are attributed to several key mechanisms:

(1) Doping of rutile (TiO_2_): Nb^5+^ ions, substituting Ti^4+^ ions in the rutile crystal lattice, reduce the concentration of oxygen vacancies. This mechanism, known as doping, significantly slows down the diffusion of oxygen into the material, limiting the scale growth rate [4,5].

(2) Increase in aluminum activity: The presence of niobium in the alloy increases the thermodynamic activity of aluminum. As a result, the critical Al concentration necessary to form a continuous, protective Al_2_O_3_ layer on the surface is lowered [3,4,6].

(3) Formation of barrier layers: Niobium promotes the formation of dense nitride (e.g., TiN) and intermetallic phase (e.g., AlNb_2_ aluminides) layers at the scale/alloy interface. These phases act as an effective diffusion barrier, inhibiting both the transport of oxygen into the alloy and the diffusion of titanium towards the surface [7,8]. At the same time, niobium introduced together with silicon limits the formation of protective titanium silicides [9] and also increases resistance to hot corrosion [10]. Microadditions to high-niobium alloys (W, B, Y, C, and Si) promote grain refinement and the formation of aluminum oxide [11].

The introduction of tungsten also leads to a significant improvement in oxidation resistance through the stabilization of a dense and compact Al_2_O_3_ layer. Its mechanism of action involves the incorporation of W ions with a high oxidation state (e.g., W^6+^) into the growing oxide scale. Similar to niobium, this modifies the defect structure of the crystal lattice, reducing anionic diffusion of oxygen and slowing down scale growth [12].

Tantalum acts similarly to Nb and in some aspects is even more effective. Ta more efficiently inhibits diffusion and more effectively promotes the formation of a continuous and dense nitride layer at the oxidation front. This results from stronger Ta-O and Ta-N bonds compared to analogous bonds involving Nb, which translates into higher thermodynamic stability of the formed protective phases [4].

Molybdenum lowers the solubility of TiO_2_, which promotes the external growth of the oxide layer and also promotes the formation of a defect-free, protective Al_2_O_3_ layer, inhibiting further oxidation [5,13]. Despite improving the mechanical properties of the alloy [14], it however worsens the resistance to hot corrosion [15,16].

The influence of chromium on oxidation resistance depends on its content in the alloy. In the concentration range of 1–3 at. %, chromium promotes the formation of titanium-rich nitrides and consequently the growth of a mixed, porous TiO_2_/Al_2_O_3_ scale with poor adhesion to the substrate [17]. At higher concentrations (>8–10 at. %), the so-called “chromium effect” is revealed, which consists of the kinetically favored oxidation of Cr in the initial stages of the process. This creates numerous nuclei for a fine-grained, compact, and protective (Al,Cr)_2_O_3_ oxide layer, which effectively inhibits the further growth of undesirable TiO_2_ [18]. The chromium present in alloys with Nb improves their resistance to hot corrosion [19].

Silver improves oxidation resistance only at lower temperatures (e.g., 800 °C) by stabilizing the so-called Z phase beneath the oxide layer. Its protective effect disappears at higher temperatures (approx. 880 °C) due to the formation of phases with a low eutectic temperature [7,20,21,22]. Silver lowers the Cr concentration necessary to obtain an aluminum oxide layer [17].

Silicon is also classified among the elements improving oxidation resistance, which is generally associated with promoting the formation of Al_2_O_3_ and improving the integrity of the scale [7,23]. A synergistic positive interaction of Si with Nb introduced by ion implantation has also been observed [24]. SiC or graphene oxide also improve oxidation resistance [25].

Tin is an element that influences the oxidation resistance of TiAl alloys through the simultaneous creation of a diffusion barrier and mechanical strengthening, which significantly improves the adhesion of the scale to the substrate [26,27].

Yttrium shows a strong dependence of its effect on concentration: Small additions (~0.3 at. %) significantly improve oxidation resistance, especially under thermal cycling conditions. The mechanism involves the formation of an internal, yttrium-rich oxide layer, composed of (Al,Y)O-type oxides (e.g., Y_2_O_3_ and Al_5_Y_3_O_12_), which increases scale adhesion. Additionally, yttrium refines oxide grains, slowing down diffusion along grain boundaries [23]. Higher concentrations (>0.6 at. %) can be harmful, leading to accelerated oxidation, particularly under isothermal conditions.

Gadolinium improves the oxidation resistance of alloys with Cr and Nb under conditions involving water vapor [28].

Vanadium, similar to nickel superalloys, is decidedly considered harmful to the oxidation resistance of TiAl alloys. Its negative effect results from the formation of volatile oxides (e.g., V_2_O_5_) with a low melting point (approx. 674 °C). Manganese and zirconium improve corrosion resistance in conditions with additional water vapor [29].

## 4. Protective Coatings for TiAl Intermetallics

### 4.1. Halogen Effect and Conversion Coatings

The oxidation resistance of γ-TiAl alloys can be significantly improved by the so-called “halogen effect”, achieved by introducing small amounts of halogens (Cl, Br, I, or F) into the alloy surface, e.g., by ion implantation or gas fluorination [30]. The interaction with halogens induces a change in the oxidation mechanism from rapid growth of a non-protective, mixed TiO_2_/Al_2_O_3_ oxide layer to the formation of a continuous, protective α-Al_2_O_3_ layer [31]. This mechanism is based on the selective formation of volatile aluminum halides (e.g., AlCl and AlF) and their subsequent oxidation within the inner parts of the scale, which leads to preferential transport and deposition of Al_2_O_3_ [32]. Thermodynamic calculations define, for each halogen, a range of partial pressures (a “window”) within which the effect is beneficial, ensuring sufficient aluminum transport while avoiding volatile titanium halides [33]. The “halogen effect” is visible under all oxidation conditions, both cyclic and isothermal, as well as under gas corrosion conditions [30]. The halogen effect can be combined with a TBC coating [34]. The results of these studies were confirmed in the case of chlorides [35].

A similar effect involving the anodizing of the Ti48Al2Nb2Cr alloy in an electrolyte containing the NH_4_F halide promoted the improvement of oxidation resistance through the formation of a layer composed of Al_2_O_3_ and TiO_2_ and fluorides (AlF_3_, TiF_4_, and CrF_3_) [36,37]. A similar effect was obtained in the fluorination process [38] or treatment with phosphoric acid [39] and manganese chloride [40]. It is also possible to combine the aluminizing process with anodizing and pre-oxidation [41]. Shot peening with aluminum oxide [42] and pre-oxidation alone also improve oxidation resistance [43]. Aluminosilicate Al_2_TiO_5_ coatings, in turn, are formed as a result of plasma oxidation in a silicate electrolyte [44].

### 4.2. Aluminide Coatings

The basic way to increase the oxidation resistance of TiAl alloys is to obtain a layer containing an increased amount of Al [45]. The main goal is to enrich the surface with sufficient aluminum to change the oxidation mechanism from the formation of a non-protective mixture of TiO_2_ and Al_2_O_3_ oxides to the formation of a continuous, dense, and compact Al_2_O_3_ layer [46,108].

Various techniques are used to produce simple aluminide coatings. The basic and most common method is halide-activated pack cementation, in which samples are annealed in a powder mixture containing an aluminum source (e.g., Al powder), an activator (e.g., NH_4_Cl or AlF_3_), and an inert filler (e.g., Al_2_O_3_) [76,109,110,111,112,113]. It is important to select an appropriate activator that ensures the formation of an aluminide layer because some of them do not lead to its formation [113,114]. At high temperatures, the activator reacts with the Al source, forming volatile halides, and the coating forms as a result of aluminum diffusion into the substrate from the vapor phase [46,77,78,79,80]. Another method is Vapor Phase Aluminizing, in which Al-Cr granules are used instead of powder [78,81,82]. This method was used to manufacture prototypes of turbine blades from TiAl alloys with this coating [79]. Work has also been carried out on the application of aluminizing by the CVD method, which is however difficult due to the low activity of Al in this process [82]. In extreme cases, it may even lead to dealuminization of the substrate material [83]. The influence of powder composition in aluminizing processes of modern TiAl alloys was studied by Grüters and Galetz [113] in the range of Al content from 20 to 100 wt.%.

The production of aluminide coatings is also possible in a two-step process involving thermal spraying with diffusion treatment. In the first step, a layer of pure aluminum is deposited, for example, by arc spraying or cold spray [115], and then a diffusion heat treatment is carried out at a high temperature (e.g., 1100 °C). This leads to the formation of intermetallic layers as a result of the diffusion reaction between the thermally sprayed Al coating and the substrate [116]. Additionally, the oxidation resistance of such a coating can be improved by obtaining a TiAl_3_/Al_2_O_3_ composite coating [117].

On the other hand, the Electro-Spark Deposition (ESD) technique uses high-energy electrical discharges between an electrode made of pure aluminum and the TiAl substrate. The high temperature of the spark causes partial melting and mixing of the materials, which, after rapid solidification, creates an aluminum-enriched surface layer that is metallurgically bonded to the substrate [77].

Obtaining high-aluminum coatings is possible by combining the deposition of a suitably thick (about 20 μm) Al layer and its diffusion treatment [116]. Another solution is the deposition of a TiAl_2_ coating by magnetron sputtering, which also leads to the formation of a protective aluminum oxide layer [47]. Increasing the heat resistance of Al-rich TiAl coatings deposited by magnetron sputtering achieves particularly good results on alloys containing elements such as niobium [48,49,50]. Miyake et al. [118] applied an electrochemical deposition of Al in a dimethylsulfone bath and diffusion annealing.

The microstructure of simple aluminide coatings depends on the deposition method used. On simple TiAl alloys (e.g., Ti-50Al), the powder method typically leads to the formation of a two-layer structure, consisting of an outer TiAl_3_ layer and an inner TiAl_2_ layer [110]. Thermal spraying with diffusion treatment results in the formation of a multilayer structure with an aluminum concentration gradient, which includes an outer, porous TiAl_3_ layer and an intermediate zone containing Ti_2_Al_5_ and TiAl_2_ phases and aluminum-rich TiAl [119]. Electro-spark deposition, on the other hand, creates a coating with a gradient structure, where the outer part has a composition close to stoichiometric TiAl_3_, and the aluminum concentration gradually decreases towards the substrate [77].

All these coatings significantly improve oxidation resistance compared to uncoated alloys because they promote the formation of a dense α-Al_2_O_3_ oxide layer instead of porous TiO_2_. A drawback of such layers, composed, for example, of the brittle TiAl_3_ phase formed on binary alloys, is susceptibility to cracking, which can lead to local loss of protective properties and enable oxygen access to the substrate [46,76]. Elements diffusing from the substrate material also influence the improvement of oxidation resistance of aluminide layers. The addition of small amounts of chromium or niobium to the TiAl alloy significantly modifies the aluminizing process and the properties of the resulting coating [6]. Compared to binary substrates, accelerated coating growth kinetics are observed on alloys with the addition of Nb or Cr, which is related to increased diffusion of aluminum in the solid state. Moreover, instead of the two-layer structure (TiAl_3_/TiAl_2_) observed on pure TiAl, a single, thicker TiAl_3_ layer is formed [76]. The improvement in oxidation resistance is significant in this case and results not only from the formation of a thicker layer but primarily from the improvement of its mechanical properties—the ductility of the TiAl_3_ phase. The pure TiAl_3_ phase crystallizes in the tetragonal DO_22_ structure, which, due to its low symmetry, possesses few slip systems at high temperatures, making it brittle. The Nb or Cr contained in the alloys can promote a phase transformation to a more ductile, regular L1_2_ structure, which is characterized by a larger number of slip systems. This leads to the formation of fewer cracks in the coating, which prevents direct oxygen access to the substrate and significantly improves oxidation resistance compared to coatings on TiAl substrates without alloying additions [76]. On the Ti45Al5Nb alloy, a coating produced by the gas phase aluminizing method exhibited a complex, layered structure with TiAl_2_ and TiAl_3_ phases. Importantly, its inner zone consisted of columnar grains enriched in niobium (up to 10 at.%), which diffused from the substrate. Such a coating enabled the formation of a thin, dense, and continuous aluminum oxide layer that did not spall, even after several hundred hours of operation at 950 °C [78]. On the other hand, on the Ti-45Al-5Nb-0.2B-0.2C alloy, it was observed that during high-temperature oxidation, the outward diffusion of titanium from the substrate leads to the formation of a TiAl phase sublayer at the coating–substrate interface. Niobium-rich phases precipitate at the boundary between this sublayer and the coating. This coating provided effective, long-term protection, and the parabolic oxidation rate constant was two orders of magnitude lower than for an uncoated alloy [46]. A similar effect of yttrium and zirconium diffusion into the aluminide layer produced by the pack cementation method was observed in the case of the two-phase Ti-47Al-2Nb-2Cr-0.5Y-0.5Zr alloy [120] and for a magnetron sputtered coating, which also improved resistance to hot corrosion [121]. An improvement in oxidation resistance was achieved by introducing an additional SiO_2_ layer on the aluminized TiAl alloy [122].

### 4.3. TiAlCr Coating

Initial attempts to improve the resistance of TiAl alloys focused on aluminide coatings produced by the pack cementation method, which, however, due to the brittleness of the TiAl_3_ phase and tendency to cracking had limited effectiveness [37,38]. The kinetic measurements of such layers were studied by Nishimoto et al. [123]. A breakthrough came with the application of TiAlCr coatings deposited by physical vapor deposition methods, including magnetron sputtering [51,124] and low-pressure plasma spraying. Studies by Fox-Rabinovich et al. [18,51] explained the key role of chromium, which through the formation of transient Cr_2_O_3_ oxides in the early stages of oxidation initiates the formation of a fine-grained (Al,Cr)_2_O_3_ layer, constituting an effective diffusion barrier. TiAlCr coatings are characterized by a typical two-phase microstructure, combining the γ-TiAl phase with the thermodynamically stable Laves phase Ti(Al,Cr)_2_, which provides a favorable compromise between plasticity and strength [17,52,53,54,55,56]. In the case of exposure in complex environments, these coatings exhibit excellent resistance to hot corrosion by forming a continuous Al_2_O_3_ layer, but they degrade in the presence of chlorides [52]. An alternative production method is sulfidation processing, which leads to the formation of complex structures with the Laves phase, significantly improving scale adhesion [125]. The long-term durability of these coatings depends on the stability of the diffusion layer at the interface with the substrate, with coatings having the L1_2_ structure (Al-21Ti-23Cr) that shows better properties than γ-phase matrix coatings [53]. The process of chromizing followed by aluminizing also allows for obtaining a more ductile Ti(Al,Cr)_3_ phase [126]. Promising results were also obtained for TiAlCr coatings produced by the plasma chromizing technique, which promote preferential oxidation of aluminum [127]. In studies by Pilone et al. [128], it was additionally shown that surface modifications, such as anodizing in phosphoric acid, can assist protection against oxidation.

### 4.4. Advanced Multicomponent Coatings

Further research on TiAlCr-type coatings focused on introducing additional elements to improve the adhesion and stability of the oxide layer at higher temperatures. The addition of reactive elements, such as Y and Si, to TiAlCr coatings deposited by PVD or vacuum plasma spraying (VPS) methods, significantly improved their protective properties. Yttrium, segregating at the grain boundaries of the Al_2_O_3_ oxide layer, mechanically strengthens its adhesion to the substrate [57], and TiAlCrY coatings deposited by the VPS method provide excellent oxidation resistance even at extreme temperatures of 1100–1200 °C, due to their ability to rapidly form a stable α-Al_2_O_3_ layer [58]. Another solution is the modification of the TiAl coating by introducing gold, which also improves corrosion resistance, especially under hot corrosion conditions [59,60].

The development of TiAlCrY-based coatings represents a significant advancement in the protection of γ-TiAl alloys against high-temperature oxidation. These coatings are typically deposited by methods such as (CHC-PVD) and Vacuum Plasma Spraying (VPS), which enables the production of dense, adherent layers with a controlled microstructure [58,61]. After deposition, the coating often exhibits a multiphase structure, including the Laves phase Ti(Al,Cr)_2_ and γ-TiAl, which evolves under thermal exposure into a more oxidation-resistant, continuous layer. The addition of chromium promotes the formation of a protective α-Al_2_O_3_ layer by facilitating the nucleation of chromium-doped transient aluminum oxide in the initial stages of oxidation [62]. Yttrium plays a key role as a reactive element, segregating to the Al_2_O_3_ grain boundaries, which improves the adhesion of the oxide layer and reduces the oxidation rate [57,58]. As a result, TiAlCrY coatings exhibit excellent oxidation resistance up to 1200 °C, with low mass gain and minimal spallation, making them promising bond coats for thermal barrier coating systems on TiAl alloys [62,63].

Further progress was achieved through the development of nitride coatings. The nanostructured, multilayer CrAlYN/CrN coating, due to numerous interfacial boundaries and the formation of a compact Al_2_O_3_ aluminum oxide layer, reduced the mass gain of the alloy fourfold at 750 °C [64,65].

In turn, TiAlSiN and TiAlSiCN coatings currently represent some of the most promising protective materials for TiAl-based alloys used in high-temperature environments. The addition of silicon in the TiAlSiN structure promotes the formation of a continuous Al_2_O_3_ layer during oxidation, which significantly improves oxidation resistance compared to conventional TiAlN coatings [66]. Moreover, the presence of Si leads to the formation of a Ti_5_Si_3_ silicide diffusion barrier at the coating–substrate interface, limiting mutual diffusion of elements and thus stabilizing the coating during long-term exposure at 900 °C [67]. In multilayer coatings, such as SiBCN/TiAlSiCN and AlOx/TiAlSiCN, additional amorphous SiBCN or AlO_x_ layers effectively limit recrystallization and diffusion processes, increasing oxidation resistance up to temperatures even above 1100 °C [68,69]. The high thermal stability of these coatings results from the presence of nanocrystalline (Ti,Al)(C,N) phases surrounded by amorphous Si-C-N interlayers, which act as diffusion barriers for oxygen [70]. As a novel solution, coatings based on the MAX-phase Cr_2_AlC have also been proposed, which improve oxidation resistance in the range of 700–800 °C by forming an Al_2_O_3_/Cr_2_O_3_ layer; however, their limitation is the diffusion of aluminum into the substrate, leading to coating depletion and the formation of brittle carbide phases [68,69,129].

The described coatings can be combined with thermal barrier coatings. An example is the three-layer YSZ/TiAlCrY/TiAlCrNb system produced by the APS/VPS method, in which the introduction of an additional, ductile TiAlCrNb buffer layer between the brittle bond coat and the substrate nearly doubles the lifetime of the system during thermal cycling at 1100 °C [62]. On the other hand, the combination of a TiAlCrYSi bond coat with a YSZ top coat deposited by the EB-PVD method is presented in [61].

### 4.5. SiBCN Coatings

Coatings based on Si-B-C-N exhibit very good properties in terms of protecting TiAl intermetallic substrates against oxidation processes. Their good properties result from strong covalent bonds, as well as a favorable combination of mechanical properties, high thermal stability, and excellent oxidation resistance at elevated temperatures, which is why they are readily used in demanding conditions. These coatings are characterized by an amorphous and disordered structure, which results in the absence of interfacial boundaries and a minimal number of paths conducive to oxygen diffusion. A material with such a disordered structure, lacking interfacial boundaries, is characterized by a limited number of paths conducive to oxygen diffusion. This guarantees the high protective quality of such coatings operated at high temperatures. The network of interatomic bonds contributes to significant inhibition of oxygen diffusion as well as chemical stability and high adhesion of the layers to TiAl intermetallic alloys [130]. Despite the known protective properties, relatively little data is available in the literature concerning the analysis of the behavior of Si-B-C-N layers as oxidation-resistant coatings deposited on an intermetallic substrate under conditions corresponding to real applications, i.e., during long-term exposure of the surface layer to temperatures close to 1000 °C. Most mechanical and material analyses were based on short time intervals of tests or standard substrates, which limited the assessment of long-term durability. Research aimed at determining properties over a longer-time perspective was carried out by Simova et al. [130] They showed that coatings obtained by DC and RF magnetron co-sputtering from Si and B_4_C targets in Ar + N_2_ gas mixtures can form continuous amorphous Si-B-C-N layers with high oxidation resistance at temperatures close to 1000 °C [130]. Earlier studies showed that SiBCN coatings maintain an amorphous structure even at extreme temperatures of up to 1700 °C, exhibiting minimal mass gain and high phase stability [131]. Microstructure studies also helped to establish that the interdiffusion of Si and Ti in the coating–substrate interfacial zone contributes to increased coating adhesion and limits thermal stresses [132]. Additionally, SiBCN coatings are characterized by high hardness, low thermal conductivity, and a beneficial influence of nitrogen composition on electronic properties, which positively affects the improvement of thermal stability and oxidation resistance [108]. In the case of multilayer coating systems, e.g., SiBCN/TiAlSiCN, the SiBCN coating constituted a stable diffusion barrier, improving the resistance of the entire coating system [70]. Si-B-C-N coatings deposited on intermetallic alloys are characterized by high amorphous stability, as well as effective anti-oxidation protection and good adhesion. Therefore, such layers are among the most promising protective solutions in high-temperature applications.

### 4.6. Mo-Si-Ti Coatings

Mo-Si-Ti coatings deposited on γ-TiAl alloys belong to materials protecting the substrate against oxidation at high temperatures. They exhibit excellent mechanical properties and high wear resistance, making them very good protective materials for TiAl intermetallic substrates [133]. Both the microstructure and the mechanical and functional properties of such coatings were studied. Produced Mo-Si layers showed promising mechanical properties and high efficiency at elevated temperatures; however, it was found that molybdenum oxides formed at higher temperatures can cause structural defects in the form of oxide-layer cracking and decomposition of the metallic material into powder form. The Mo-Si-Ti coating with a gradient structure contained (Ti,Mo)_5_Si_3_, MoSi_2_, and TiSi phases, and its hardness was several times higher than that of the uncoated γ-TiAl substrate. Due to increased H/E and H^3^/E^2^ parameter values, the coating exhibited greater resistance to plastic deformation and load-bearing capacity, and tribological wear decreased by up to 98% compared to the substrate [133]. The production of Mo-Si coatings with the addition of titanium by the DGP (Double Glow Plasma) method was also considered, aiming to limit problems related to oxide-layer cracking. During oxidation at 750 °C, the coating forms a stable, fine-grained protective SiO_2_/TiO_2_ layer, which limits oxygen diffusion to the substrate and prevents oxide-layer spallation, increasing the resistance of the γ-TiAl alloy [134]. Two-phase MoSi_2_/(Mo,Ti)Si_2_ coatings form mixed Si-Ti-O oxides, effectively protecting against oxidation [135]. Additionally, it was found that wear-resistant Mo-Si-Ti coatings contain cracks resulting from the difference in Young’s modulus between the intermetallic substrate and the coating. A promising solution to counteract this drawback is the production of coatings with a gradient structure, achieving a change in structure or properties in one or multiple directions. Functionally gradient MoSi_2_/Mo coatings improve adhesion, reduce thermal stresses, and extend the protective life of the coating [136]. In summary, Mo-Si-Ti coatings on γ-TiAl are characterized by high hardness, a stable microstructure, and the ability to form protective oxide layers, which can significantly extend the material’s durability in high-temperature operating conditions and tribological applications.

### 4.7. Aluminum-Based Coatings with Silicon

The main goal of introducing silicon into aluminide layers on γ-TiAl alloy substrates is the synergistic action with aluminum to produce a stable, continuous aluminum oxide (Al_2_O_3_) layer and simultaneously inhibit the formation of undesirable, rapidly growing titanium oxides (TiO_2_). Silicon plays a dual role here: firstly, it acts as a so-called “getter” for titanium, binding it into thermodynamically stable silicide phases (e.g., Ti_5_Si_3_), which limits the availability of titanium for reaction with oxygen. Secondly, the presence of silicides in the coating structure can create additional diffusion barriers, slowing down the migration of elements between the coating and the substrate and delaying the process of aluminum depletion in the coating [137].

The basic method for obtaining aluminum and silicon-based layers is pack cementation [138]. Research conducted by Xiang et al. [139,140] focused on thermodynamic analysis as well as experimental processes using this method with various halide activators. The produced coating had a complex, multilayer structure, consisting of an outer silicide layer (containing phases such as Ti_5_Si_4_, Ti_5_Si_3_, TiSi, and TiSi_2_) and an inner aluminum TiAl_3_ layer, separated by a transition zone. The coating showed good oxidation resistance at 850 °C for 240 h, confirmed by a small mass gain. The protective scale consisted of a mixture of Al_2_O_3_, SiO_2_, and TiO_2_ oxides, and the coating itself proved thermally stable at this temperature.

The powder method (pack cementation) for producing Si-aluminide coatings with low and high silicon content was used by Swadźba et al. [141]. The obtained coatings had a structure consisting of an outer TiAl_3_ zone and an inner TiAl_2_ zone, both containing nanometric titanium silicide precipitates. Long-term (3013 h) cyclic oxidation tests at 850 °C showed a significant improvement in oxidation resistance and a very low mass gain of the samples. The scale mainly consisted of a continuous and thin α-Al_2_O_3_ layer, and during exposure, segregation and growth of Ti_5_Si_3_ precipitates were observed at the metal–scale interface, which further enhanced the barrier properties. Further studies of corrosion resistance at 950 °C confirmed its high heat resistance also under such conditions [142]. Similar results were obtained by Wozniak et al., and numerous porosities were found in the obtained layers [143,144].

The slurry method is one of the most cost-effective ways to produce diffusion protective coatings on geometrically complex components made of TiAl alloys [105]. This process involves applying an aqueous slurry to the component surface, drying it, and diffusion annealing, which is typically carried out at a temperature of about 950 °C in a protective argon atmosphere. During diffusion annealing, silicon reacts with titanium diffusing from the substrate, leading to the formation of thermodynamically stable titanium silicides (Ti_x_Si_y_). As a result of these processes, the aluminum–silicon coating on the TiAl alloy acquires a characteristic, multi-zone structure, which can be described as follows [106]: (1) The outer zone consists of a TiAl_3_-phase matrix, in which fine, dispersed silicide precipitates are observed, often located at grain boundaries. (2) The middle zone is characterized by the presence of columnar silicide grains (mainly Ti_5_Si_3_) embedded in a TiAl_3_-phase matrix. (3) The inner zone is a thin, continuous layer of the TiAl_2_ phase.

The type and amount of silicides formed depend on the Si concentration in the slurry. At lower concentrations (e.g., 5–12.5 at. % Si), phases such as Ti_5_Si_3_ and Ti_5_Si_4_ are identified in the coating. An increase in silicon content (above 20 at. % Si) additionally leads to the formation of TiSi_2_ silicide [107].

In turn, Xiong et al. [101,102] produced a coating by immersing a TiAl sample in the molten Al-Si alloy (liquid-phase siliconizing). The layer thus formed consisted of the ternary Ti_7_Al_5_Si_12_ phase and binary silicides TiSi_2_ and Ti_5_Si_4_. During oxidation at 900 °C, the coating significantly improved the alloy’s resistance, and its scale was a mixture of Al_2_O_3_, TiO_2_, and SiO_2_ oxides. Importantly, during the oxidation process, additional diffusion bands composed of Ti_5_Si_4_ and TiAl_2_ formed at the interface, which acted as additional barriers to diffusion [103,104].

Similar to simple aluminide layers, it is possible to obtain Al-Si layers by combining the process of thermal spraying of Al-Si powder, e.g., plasma [144] or cold spray [91,92], with subsequent diffusion annealing, which leads to the formation of a coating similar in structure to those obtained by pack cementation and slurry methods. Huang [93] demonstrated that cold-spray deposition of powder containing 40 wt.% Si causes, during oxidation, the formation of a diffusion barrier composed of Ti_5_Si_3_.

Research conducted by Moskal et al. [71] concerned diffusion of Al-Si coatings deposited by the Arc-PVD method on a TiAlCrNb alloy. The process consisted of two stages: first, an AlSi layer was deposited, and then it was annealed in a vacuum to form a diffusion coating with a multilayer structure. It consisted of sublayers, including TiAl_3_ (with silicon, chromium, and niobium in solid solution) and Ti-Si intermetallic phases, which promoted the formation of an aluminum-enriched inner zone. In cyclic oxidation tests at 950 °C, these coatings effectively reduced the degradation rate, especially during the first 40 cycles, due to the formation of a protective Al_2_O_3_ layer [72].

Similar results at 950 °C, but using a different physical vapor deposition technique (HS-PVD), were obtained by Bobzin et al. [73], who applied this method to deposit Al-Si coatings on a γ-TiAl alloy, obtaining crack-free layers with good adhesion. Analysis of the coating with a composition of 79% Al and 21% Si during cyclic oxidation at 950 °C showed its transformation into intermetallic phases, such as Al_3_Ti. After 150 cycles, the main phases providing protection became α-Al_2_O_3_ and stable Ti_5_Si_3_ silicide. The authors emphasized that the addition of silicon significantly improved crack resistance and thermomechanical compatibility at the scale–coating interface, effectively binding titanium in the form of Ti_5_Si_3_ and thus limiting the formation of undesirable TiO_2_.

In the work by Bauer et al. [74,75,141], an Al-18Si (at.%) coating deposited by DC magnetron sputtering was analyzed. In the as-deposited state, the coating was characterized by a crystalline microstructure of Si grains in an Al matrix, which, after heat treatment (550 °C, 20 h), completely transformed into the TiAl_3_ phase. During isothermal oxidation at 850 °C, the coating formed a protective α-Al_2_O_3_ layer, and silicon played a dual role. This confirms the dual role of silicon, also identified by Bobzin et al. [73], where the Ti_5_Si_3_ phase not only binds titanium as a “getter,” preventing its oxidation to TiO_2_, but also acts as a diffusion barrier, limiting the migration of aluminum to the substrate and thus slowing down coating degradation. The addition of yttrium also had a positive effect. Coatings containing Al and Si can also be a part of composite coatings, e.g., glass/Ti_5_Si_3_/α-Al_2_O_3_/Ti_3_Al [95] or aluminum–SiO_2_ [96,97].

### 4.8. Silicon and Silicon-Based Coatings

Silicide coatings represent an alternative to aluminum-based systems and are characterized by an equally effective mechanism of protection against high-temperature oxidation of TiAl alloys. Their action relies on the formation of thermodynamically stable silicon oxide (SiO_2_) layers on the alloy surface, characterized by a very slow growth, during oxidation. These layers, often amorphous in nature, act as a diffusion barrier, inhibiting oxygen transport towards the substrate. Depending on the coating composition and oxidation conditions, mixed oxide layers (e.g., Al_2_O_3_-SiO_2_) may also form, combining the advantages of both protective systems. Thin layers of Ti5Si3 silicide were obtained by Gray et al. as a result of heat treatment of a near-γ-TiAl alloy in silica capsules [145].

Studies by Chaia et al. [111] focused on MSi_2_-type silicide coatings (where M = Nb or Ti) produced on a Nb-Ti-Al alloy by the powder method (HAPC) with a BaF_2_ activator. The resulting coating consisted of a thick (approx. 60 μm) outer MSi_2_ silicide layer and an interdiffusion zone with aluminum-rich precipitates. Oxidation tests in the range of 900–1000 °C confirmed the high resistance of the coating, which formed a protective SiO_2_ scale with dispersed TiO_2_ particles. Its effectiveness was evidenced by the very low mass gain value, amounting to only about 1.2 mg·cm^−2^ after 670 h of exposure at 1000 °C. Another concept for silicide coatings was proposed by Abu Sulik et al. [146], introducing refractory metal silicides, among which niobium silicides proved effective.

Rubacha et al. [147] developed a silicon-rich coating on a Ti-46Al-8Ta alloy, applying a two-step process: first, magnetron sputtering of a Ti-10Si layer, followed by powder deposition. The obtained thick (approx. 60 μm) and multiphase coating consisted of TiSi_2_/TiSi and Ti_5_Si_4_/Ti_5_Si_3_ layers and an aluminum-enriched zone (TiAl_2_). The coating showed significantly better resistance under high-temperature corrosion conditions (800 °C with salt deposits), which was attributed to the formation of an oxide layer composed of amorphous silica with embedded rutile and cristobalite crystals, which constituted an effective barrier to oxygen.

The role of silicon in the TiAlSiN coating during long-term oxidation at 900 °C was the subject of analysis by Zhang et al. [67]. The key element of the protective mechanism turned out to be the formation of a stable Ti_5_Si_3_ diffusion layer at the coating/substrate interface, resulting from the inward diffusion of silicon. This layer effectively inhibited nitrogen diffusion, ensuring the long-term stability of the entire coating. Additionally, a protective Al_2_O_3_ layer formed on the surface, and SiO_2_ segregation at grain boundaries and at the scale/coating interface inhibited oxygen and titanium diffusion.

Li et al. [148] described yttrium-modified silicide coatings deposited by the pack cementation method. They were characterized by a complex, multilayer structure, consisting, among others, of (Ti,Nb)_5_Si_4_, (Ti,Nb)_5_Si_3_, and (Ti,Nb)Si_2_ phases. In an isothermal oxidation test at 1000 °C, the coatings showed good resistance, and the oxidation rate constant was two orders of magnitude lower than that of the base alloy. After 50 h of exposure, a dense scale composed of SiO_2_, Al_2_O_3_, and TiO_2_ formed on the surface. On the other hand, the positive effect of the synergistic interaction of a magnetron-sputtered Pt and Si coating through the formation of Pt_x_Si_γ_, Pt_x_Al_γ_, and Ti_x_Si_γ_ phases was demonstrated by Crespo-Villegas et al. [149].

A composite coating produced by powder siliconizing in a mixture of 15% Si and 85% Al_2_O_3_ was presented by Liang et al. [84]. Its structure consisted of an inner Ti_5_Si_3_-based layer and an outer Al_2_O_3_-based layer. The results of cyclic oxidation at 900 °C indicate that the resistance increased with the siliconizing process temperature. Exceptional effectiveness was shown by the coating obtained at 1250 °C, which, after 1000 h of testing, recorded a mass gain below 0.3 mg cm^−2^, which the authors attributed to the stability of the Ti_5_Si_3_ layer and the density of the outer Al_2_O_3_ layer.

In the work by Crespo-Villegas [85], Ti_x_Si_y_ coatings were produced by magnetron sputtering of a silicon layer, followed by vacuum annealing at 950 °C. The resulting multi-zone structure consisted of an outer titanium silicides coating, an interdiffusion zone, and an inner TiAl_2_ zone. During oxidation at 900 °C, the coatings provided effective protection. The scale composition depended on the initial Si layer thickness: for thinner layers, silicon and titanium oxides dominated, while for thicker ones, a dense mixture of silicon and aluminum oxides formed with significant chromium enrichment.

A key role in ensuring long-term protection at high temperatures by silicide coatings is played by the controlled formation of stable phases, such as Ti_5_Si_3_, which act as diffusion barriers, and the formation on the surface of protective silicon oxide (SiO_2_) layers, often in combination with Al_2_O_3_. Additionally, the SiO_2_ layer improves resistance to hot corrosion. Similar to Al-Si coatings, silicide coatings can also be used as composites, e.g., Ti_5_Si_3_/γ/TiSi [94], even with the addition of hafnium as HfSi_2_-HfO_2_-SiO_2_ [150].

### 4.9. NiAl-Based Coatings

NiAl coatings, used for decades to protect nickel superalloys, can also be applied on TiAl alloys [86]. They are mainly produced by a two-step method, consisting of nickel deposition, e.g., electrochemically, followed by aluminizing [87]. The use of processes with high aluminum activity (using Al powder) at 1273 K leads to the formation of a multilayer diffusion structure, characterized by an outer δ-Ni_2_Al_3_ layer and inner TiNiAl_2_, TiAl_3_, and TiAl_2_ layers, with the inner layers containing more aluminum than the outer one [88,89,90]. These coatings exhibit long-term oxidation resistance at 1173 K, forming a protective Al_2_O_3_ scale with minimal spallation and mass gain on the order of 37 g/m^2^ after 36,000 ks [88]. A key advantage is the phenomenon of “uphill diffusion”, which ensures a higher aluminum content in the inner layers, constituting a reservoir for the regeneration of the protective oxide. The main drawback is the formation of porosity in the outer layer during the δ-Ni_2_Al_3_-to-β-NiAl phase transformation, which can potentially affect the mechanical integrity of the coating [88,89,151]. Alternatively, vacuum plasma spraying (VPS) allows for the deposition of a β-NiAl layer, but it involves the formation of a ternary intermetallic layer (e.g., AlTiNi-type phases) in the interdiffusion zone with the substrate, which may affect the brittleness of the bond [152]. Platinum-modified aluminide coatings [153], deposited by sputtering, were also studied, showing very low mass gain (below) after 1500 cycles on alloys [154,155]. Besides those described, attempts are being made to apply 80Ni020Cr coatings [156] as well as introduce Ni through plasma carburizing [157].

### 4.10. MCrAlY Coatings

MCrAlY-type coatings (where M = Ni or Co) are deposited on TiAl alloys by various methods, including plasma spraying and magnetron sputtering. Due to their aluminum content, during high-temperature oxidation, a protective Al_2_O_3_ layer forms on the coating surface, which significantly improves cyclic oxidation resistance [158]. Similar to these coatings deposited on nickel superalloys, apart from pure chromium or aluminum oxides, spinels such as CoCr_2_O_4_ and NiCr_2_O_4_ may form on their surface [159]. However, these coatings are characterized by poor compatibility with the TiAl substrate, leading to intensive interdiffusion and the formation of brittle intermetallic phases (e.g., AlNi_2_Ti or AlCo_2_Ti) and Kirkendall voids, which reduces the coating’s durability and the mechanical properties of the substrate [101,160,161]. This problem is solved by, among other methods, using diffusion barriers. It has been shown that the application of an intermediate Cr_2_O_3_ layer, produced by arc ion plating, effectively inhibits this diffusion by forming continuous, Al_2_O_3_-rich layers, acting as an active diffusion barrier [162]. A metallic Mo barrier effectively slowed down diffusion, reducing the thickness of the interdiffusion zone even fivefold compared to a coating without a barrier [163,164]. Alternatively, Ta addition can also be used [165]. Significantly better results were achieved using a ceramic layer of the MAX-phase Cr_2_AlC, which, during operation, transformed into Cr_2_C_3_, α-Cr, and TiC layers, effectively blocking titanium diffusion [166]. Studies by Tian et al. [167] directly demonstrate the negative consequences of lacking a barrier, where intensive diffusion led to the formation of a brittle interdiffusion zone (IDZ) with Al-Ni-Ti phases and to the segregation of a TiN phase initiating microcracks, which drastically shortened the coating’s lifetime.

The 8YSZ/NiCoCrAlY TBC coating significantly improves the oxidation resistance of the TNM alloy at high temperatures, reducing the mass gain after 250 h at 950 °C from 6.98 mg/cm^2^ to 2.45 mg/cm^2^. Ti-Al-N- and Nb-Mo-rich layers block diffusion, but over time, the protective, dense Al_2_O_3_ layer transforms into mixed CS oxides (chromia–spinel, i.e., Cr_2_O_3_ and (Ni,Co)Al_2_O_4_) and subsequently into CSN (chromia–spinel–nickel oxide, i.e., Cr_2_O_3_, (Ni,Co)Al_2_O_4_, and NiO), which leads to cracking and degradation of the coating [168].

### 4.11. Characteristics of TBC and Ceramic Coating Systems Based on γ-TiAl Alloys

The key methods for producing thermal barrier coatings for TiAl alloys are atmospheric plasma spraying (APS), high-velocity oxy-fuel (HVOF) spraying, e.g., respectively for 8YSZ layers and NiCoCrAlY bond coats [168,169], as well as (EB-PVD) and PS-PVD, allowing for the formation of columnar structures in 7YSZ or YSZ layers [65,170,171]. For bond coats of the CrAlYN and Ti-Al-Cr type [65,172], PVD methods are used, as well as chemical and electrochemical methods, such as cathodic plasma electrolytic deposition (CPED) or ultrasonic spray pyrolysis, which enable the in-situ formation of ceramic layers (e.g., Al_2_O_3_-ZrO_2_ or (Al_2_O_3_--Y_2_O_3_)/YSZ laminates) from precursor solutions [173,174,175]. Furthermore, specialized PVD techniques are used, such as arc-ion plating for depositing TiAlSiN coatings with enhanced stability [66].

Similar to nickel superalloys, the basic material forming TBC coatings on TiAl alloys is yttria-stabilized zirconia, most often in the 8YSZ [169,176] or 7YSZ variants [34,65,170,177]. In the case of conventional plasma-sprayed YSZ coatings, its nanostructured nature yields good results [178]. Techniques such as EB-PVD and PS-PVD are intentionally used to create a columnar structure [171,172], which provides high strain tolerance resulting from the mismatch of thermal expansion coefficients between the ceramic layer and the metallic substrate. Alternative concepts rely on abandoning the traditional multilayer TBC construction in favor of single or laminated ceramic coatings that function as an oxidation barrier themselves, e.g., composed of Al_2_O_3_ [179]. Examples are lamellar (Al_2_O_3_-Y_2_O_3_)/YSZ layers produced by spray pyrolysis, which, due to the formation of an interlocking interlayer structure, exhibit better mechanical properties [174], or single, dense Al_2_O_3_ layers [180,181] which can be doped with Au particles [182] or ZrO_2_/YSZ deposited by the CPED technique [173], as well as Al_2_O_3_-ZrO_2_ coatings themselves [183]. Li et al. [184] on the other hand, applied an amorphous composite coating composed of SiC-Al_2_O_3_. It is also possible to use Al_2_O_3_-YSZ coatings with an additional Al-Y coating, which also increases oxidation resistance [185]. Another example of ceramic coatings is the HfSi_2_-HfO_2_-SiO_2_ nanocomposite coating [150]. Regardless of the structure of such coatings, their durability is determined by the stability of the interfacial zone and, in particular, the controlled growth of the thermally grown oxide (TGO) layer. Silicon oxide [186] can also be used as an oxide layer, which can be modified with nickel [187]. Studies of Ni-SiO_2_ coatings confirmed their high oxidation resistance under both isothermal and cyclic conditions at elevated temperatures of around 900 °C. Analyses showed that the addition of nickel affects the resulting thermal stresses and prevents the initiation and propagation of cracks in the coating. Silicon oxide, SiO_2_, layers obtained by electrochemical deposition and subsequently subjected to oxidation were also analyzed. Another example of silicon-based coatings is the deposition of the SiOC coating by the sol–gel method [188], which can be modified by introducing a silicon oxide layer [98,99,189,190].

In the case of TiAl alloys with MCrAlY bond coats, degradation is driven by the depletion of the aluminum reservoir and the outward diffusion of other metallic cations (Ni, Co, and Cr). This process leads to the transformation of the initially protective Al_2_O_3_ layer into harmful, mixed external oxides, such as the CS layer (chromium oxide or spinel) and CSN clusters (chromium oxide, spinel, and nickel oxide), which generates stresses and leads to loss of cohesion [168]. In turn, in systems with nitride bond coats, degradation is initiated by the release of nitrogen and its inward diffusion into the TiAl substrate. This causes the formation of hard and brittle nitrides, such as TiN and Ti_2_AlN, at the interface [65,100,168,191,192]. An alternative type is the previously described TiAlSiN coatings, in which silicon diffuses towards the substrate, forming a diffusion barrier in the form of the Ti_5_Si_3_ phase. This layer effectively inhibits nitrogen diffusion, significantly improving thermal stability [66]. Regardless of the mechanism, unfavorable interdiffusion zones also form at the interface of the MCrAlY bond coat and the TiAl substrate, e.g., composed of Al(Ni,Co)_2_Ti phases [169]. Generally, the most important types of interlayers for TBC coatings on TiAl alloys are:

- MCrAlY-type bond coats: Due to the formation of unfavorable phases at the interface with the TiAl alloy substrate, various types of modifications are used. For example, the addition of Nb nanoparticles to NiCoCrAlY powder leads to solid solution and dispersion strengthening, as well as the formation of core–shell nano-Nb-Al_2_O_3_ structures, which significantly improves the layer’s resistance to deformation. At the same time, it stabilizes TGO growth, promoting the formation of a fine-grained Al_2_O_3_ structure [169].
- CrAlYN and TiAlSiN bond coats: Nitride-based coatings allow for achieving comparable oxidation protection. CrAlYN layers, despite gradual degradation, oxidize to a stable layer of mixed (Al,Cr)_2_O_3_ oxides, which provides very good adhesion for the YSZ top layer. Even greater thermal stability is exhibited by TiAlSiN coatings, which is related to the inward diffusion of silicon into the substrate and the formation of a Ti_5_Si_3_ silicide diffusion barrier at the interface. This layer effectively blocks nitrogen diffusion into the TiAl alloy, preventing the formation of brittle titanium nitrides [66].
- Aluminide and TiAlCr bond coats: Classic aluminide layers, especially those modified with platinum (PtAl), exhibit exceptional effectiveness [193]. Sputtered PtAl layers effectively form a homogeneous and tight protective Al_2_O_3_ layer, ensuring durability exceeding 1500 cycles at 1000 °C [170]. Layers based on intermetallic phases, such as Ti-Al-Cr, also effectively protect the substrate; however, their durability is limited by gradual depletion of aluminum and chromium [172,194]. TiAlCrY-type bond coats show good compatibility with the ceramic layer [62,63]. On the other hand, coatings deposited by the slurry method may prove too thin to provide long-term protection [171].
- Halogen effect: The application of this effect allows for, during the initial stage of oxidation, the growth of its own, strongly adherent TGO layer based on Al_2_O_3_, which functions analogously to a conventional bond coat, ensuring good adhesion of the YSZ ceramic layer [34,177].

## 5. Conclusions

TiAl intermetallic alloys have enjoyed great interest since the end of the last century and are well known as advanced engineering materials dedicated to high-temperature applications. These materials can be a good substitute for conventional nickel superalloys widely applied in the aerospace industry. They are characterized by a favorable combination of physical and mechanical properties, including low density and good strength at elevated temperatures. Alloys based on the γ-TiAl phase are among the most promising structural materials intended for operation at elevated temperatures (700–900 °C), especially in aircraft and automotive turbine components. Despite numerous advantages, their wider application is limited by insufficient resistance to oxidation and high-temperature corrosion, as well as by inadequate wear and erosion resistance and susceptibility to microstructural degradation. In the uncoated state, the oxidation mechanism of γ-TiAl involves the rapid, initial formation of a mixture of TiO_2_ and Al_2_O_3_ oxides, with the dominant role of rutile (TiO_2_) leading to the formation of a porous, poorly adherent scale of mixed character, incapable of effectively blocking oxygen diffusion into the substrate. Consequently, the key goal of coating technologies is therefore to enforce selective aluminum oxidation and stabilize a continuous α-Al_2_O_3_ layer with a significantly lower parabolic growth constant. Hence, the development of new and improved protective coatings is one of the fundamental research tasks in the field of TiAl intermetallic alloys.

In the presented review article, a classification of protective coatings dedicated to TiAl alloys was made, and the research achievements published in the scientific literature over the last decade were characterized. The simplest and most commonly used group are aluminide coatings produced by diffusion methods (pack cementation, vapor phase aluminizing, or CVD) or by thermal spraying with subsequent diffusion treatment, leading to the formation of TiAl_3_/TiAl_2_ layers [46,76]. Their main advantage is the effective promotion of the growth of a compact Al_2_O_3_ layer and a reduction in the oxidation rate constant by even two orders of magnitude compared to the uncoated alloy [46]. However, a drawback remains: the brittleness of the TiAl_3_ phase with the DO_22_ structure and the tendency to crack under thermal cycling conditions, which can initiate local scale spallation [76]. This problem is partially solved by modifying the substrate composition (Nb or Cr), which promotes transformation to a more ductile L1_2_ structure and improves thermomechanical compatibility [6]. A development of classic aluminide layers is TiAlCr and TiAlCrY coatings deposited by PVD or VPS methods, in which chromium plays a key role by initiating the formation of transient Cr_2_O_3_ oxides and the nucleation of a fine-grained (Al,Cr)_2_O_3_ layer [18]. The addition of yttrium as a reactive element improves scale adhesion through segregation to Al_2_O_3_ grain boundaries and reduction in grain boundary diffusion [57,58]. The advantages of these systems are high resistance up to 1100–1200 °C and good compatibility with TBC systems; the drawbacks are gradual depletion of Al and Cr and the possibility of forming unfavorable interdiffusion zones upon long exposure. Analogous challenges concern MCrAlY- and NiAl-type coatings, which, despite their excellent ability to form Al_2_O_3_ [67], show limited compatibility with the TiAl substrate due to intensive interdiffusion and the formation of brittle Al(Ni,Co)_2_Ti phases and Kirkendall voids [139,161]. It thus becomes necessary to use diffusion barriers (e.g., Cr_2_O_3_, Mo, or MAX-phase Cr_2_AlC), which effectively stabilize the interfacial zone [154,158]. An alternative is provided by Al-Si and silicide coatings, in which the protective mechanism relies on the synergy of Al_2_O_3_ and SiO_2_ formation and on the formation of stable Ti_5_Si_3_ phases acting as diffusion barriers. These coatings are produced by pack cementation, slurry, PVD, or immersion in liquid Al-Si [133,134,135,139], and their multi-zone microstructure (TiAl_3_/TiAl_2_ with silicide precipitates) ensures very low mass gain during long-term oxidation [139]. Silicon acts as a “getter” for titanium, limiting its oxidation to TiO_2_ and improving resistance to hot corrosion. A drawback, however, may be the porosity of the layers and the risk of aluminum depletion over long service times. In purely silicide systems (MSi_2_ and Ti_5_Si_3_), protection relies mainly on the formation of an amorphous SiO_2_ layer with very low oxygen diffusivity [74,111], although their limitation may be lower thermal compatibility with the substrate. The presented analysis considered both conventional solutions, such as aluminide and silicon coatings, and more advanced protective-layer systems formed using modern technologies. The performed analysis indicates that the effectiveness of surface protection of TiAl alloys depends on many factors, including the type of coating, optimization of its microstructure, and the topography of the substrate surface affecting the adhesion of the protective layer. Recently, nitride coatings (CrAlYN and TiAlSiN) and multilayer nanocomposite systems have also been studied, which are characterized by high hardness and favorable oxidation kinetics due to the formation of stable (Al,Cr)_2_O_3_ and Ti_5_Si_3_ layers [65,66]. At the same time, problems may arise with the formation of brittle TiN/Ti_2_AlN nitride compounds in the interfacial zone in the case of uncontrolled diffusion [160]. Currently, the subject of coating protection for TiAl intermetallic alloys is focused on modeling multilayer and gradient-layer systems that improve resistance to oxidation, wear, and thermomechanical loads. Furthermore, new-generation functional coatings are also widely studied, including layered thermal barrier coatings (TBCs) optimized for TiAl alloys, coatings with nanometric structures, and composite layers. Concepts of self-healing coatings are also being developed. Research should also be carried out with a view to integrate surface modification processes with 3D printing technologies and model coatings dedicated to third- and fourth-generation TiAl alloys. In conclusion, it should be emphasized that further development of protective coatings for TiAl alloys should be carried out interdisciplinarily, taking into account material engineering, advanced coating-formation techniques, and analyses of surface degradation processes. Research on new coating solutions will allow for the production of better coatings with high durability under demanding operating conditions. This literature review shows that the aforementioned protective coatings will continue to be the basic method enabling the practical application of TiAl alloys in modern structures subjected to high temperatures.

## Figures and Tables

**Table 1:** Methods of protective coating production for TiAl intermetallics.

Deposition Method	Advantages	Limitations	Types of Coatings Produced	Ref. No.
Magnetron Sputtering (PVD)	Precise control of chemical composition; high coating uniformity; low process temperature; good adhesion.	Limited coating thickness; high equipment cost; line-of-sight process.	MCrAlY coatings; TiAlCr coatings; multicomponent coatings (Al-Cr-Si); thin aluminide coatings.	[17,18,47,48,49,50,51,52,53,54,55,56,57,58,59,60,61,62,63,64,65,66,67]
Physical Vapor Deposition (PVD)	High coating purity; capability to deposit multilayer architectures; good adhesion.	Limited thickness; development of residual stresses; geometric limitations.	MCrAlY coatings; TiAlCr coatings; multicomponent coatings; thin Al and Al-Si coatings.	[17,18,51,52,53,54,55,56,66,67,68,69,70,71,72,73,74,75]
Chemical Vapor Deposition (CVD)	Excellent coating uniformity; suitable for complex geometries; strong substrate bonding.	High processing temperature; potential interaction with TiAl substrate; toxic precursors.	Simple aluminide coatings (NiAl and TiAl_3_); silicide and silicon-based coatings; halide-activated conversion coatings.	[30,31,32,33,46,76,77,78,79,80,81,82,83,84,85,86,87,88,89,90]
Plasma Spraying (APS/VPS/PS-PVD)	Ability to deposit thick coatings; high thermal resistance; industrial maturity.	Coating porosity; lower adhesion compared to diffusion coatings; post-treatment often required.	MCrAlY coatings; multicomponent coatings; Al-Si coatings.	[19,58,62,63,91,92,93]
Laser Cladding	Metallurgical bonding with the substrate; good control of heat-affected zone; high durability.	High cost; risk of cracking; complex process optimization.	MCrAlY coatings; NiAl coatings; Al-Si coatings; multicomponent coatings.	[86,87,88,89,90,94]
Enameling (Glass–Ceramic Coatings)	Good oxidation resistance; low cost; simple technology.	Brittleness; limited mechanical resistance.	Silicide and silicon-based coatings; Al-Si coatings.	[95,96,97,98,99,100]
Sol–Gel Processing	Low processing temperature; chemical homogeneity; compositional flexibility.	Limited thickness; shrinkage and cracking during drying; multi-step process.	Thin silicide coatings; Al-Si coatings; multicomponent coatings.	[96,97,98]
Liquid-Phase Siliconizing	Highly effective oxidation protection; formation of diffusion layers.	High processing temperature; difficult thickness control.	Silicide and silicon-based coatings; Al-Si coatings.	[84,101,102,103,104]
Ion Implantation	No significant change in surface topography; precise compositional control.	Very shallow modification depth; high cost.	Modified surface layers, leading to aluminide, silicide, or multicomponent protective systems.	[24,30,31,32,33]
Dip Coating (Slurry Method)	Simple process; low cost; suitable for complex shapes.	Limited thickness control; cracking after drying.	Silicide coatings; Al-Si coatings; multicomponent coatings.	[105,106,107]

## Data Availability

No new data were created or analyzed in this study. Data sharing is not applicable to this article.
